# Recent Progress in Transition Metal Dichalcogenides for Electrochemical Biomolecular Detection

**DOI:** 10.3390/mi14122139

**Published:** 2023-11-22

**Authors:** Sasya Madhurantakam, Georgeena Mathew, Bianca Elizabeth David, Aliya Naqvi, Shalini Prasad

**Affiliations:** Department of Bioengineering, The University of Texas at Dallas, Richardson, TX 75248, USA; sasya.madhurantakam@utdallas.edu (S.M.);

**Keywords:** transition metal dichalcogenides, point-of-care devices, electrochemical sensor, two-dimensional materials, biomarkers

## Abstract

Advances in the field of nanobiotechnology are largely due to discoveries in the field of materials. Recent developments in the field of electrochemical biosensors based on transition metal nanomaterials as transducer elements have been beneficial as they possess various functionalities that increase surface area and provide well-defined active sites to accommodate elements for rapid detection of biomolecules. In recent years, transition metal dichalcogenides (TMDs) have become the focus of interest in various applications due to their considerable physical, chemical, electronic, and optical properties. It is worth noting that their unique properties can be modulated by defect engineering and morphology control. The resulting multifunctional TMD surfaces have been explored as potential capture probes for the rapid and selective detection of biomolecules. In this review, our primary focus is to delve into the synthesis, properties, design, and development of electrochemical biosensors that are based on transition metal dichalcogenides (TMDs) for the detection of biomolecules. We aim to explore the potential of TMD-based electrochemical biosensors, identify the challenges that need to be overcome, and highlight the opportunities for further future development.

## 1. Introduction

Within the evolving realm of medical device development, interest has grown exponentially in transition metal dichalcogenides (TMDs), a class of two-dimensional materials with a wide range of electrochemical properties that are used in research areas such as nanotechnology and nanosensing. Since the discovery of graphene in 2004, which represented a major milestone in materials research due to its excellent electrical properties and value for electronic applications, researchers have eagerly sought advances in TMDs [1]. Transition metal dichalcogenides have many ideal properties, such as high response rates, a high surface-to-volume ratio, low operating voltage, and compatibility with standard manufacturing processes [2]. Due to these specific characteristics, TMDs have taken the place of traditional semiconductors in the advancing era of electrochemical devices for biosensing research. The recent surge in COVID-19 and the resulting need for rapid yet accurate detection methods have caused the demand for biosensors with real-time biomolecule detection capabilities to skyrocket [3]. TMDs are emerging as a promising factor in the development of point-of-care biosensors for real-time detection of biomolecules in various pathological disease states.

Transition metal dichalcogenides consist of the MX_2_ form, where M is the transition metal (Mn, Mo, Cr, Ti, V, Zr, Nb, Tc, Ta, Hf, W, or Re) and X is the chalcogen (O, S, Se, Te, Po) [4]. Conventionally, transition metals are preferred due to their appealing mechanical properties, but the lack of an electronic band gap led researchers to discover 2D materials that have semiconducting properties [5]. TMD materials are layered, with the plane of metal atoms sandwiched between two opposing planes of chalcogen atoms covalently bonded in the described layer and through van der Waal (VdW) interactions between successive layers [4]. These weak van der Waal interactions allow for layer-controlled synthesis, giving TMDs unique optoelectronic properties through this reduction from multilayers to monolayers. The VdW interactions can be mechanically disrupted by exfoliation techniques such as liquid-phase exfoliation, electrochemistry, and intercalation chemistry [6]. Liquid-phase exfoliation is a promising method that yields a large amount of 2D nanosheets in a wide thickness range from tens to hundreds of nanometers and attracts much attention due to its unique physical and chemical properties [7]. The X-M-X single-layer isolation of the material causes a shift in the electronic band structure from indirect to direct, which increases the likelihood of its use in transistor fabrication due to its strong photoluminescence and large exciton binding energy [8]. The finite band gap in semiconductor materials such as TMDs is important for the research and development of electrochemical biosensors. When 2D transition metal dichalcogenides are atomically diluted into a single layer, the electrical structure shifts from an indirect to a direct band gap, which is of interest for electrochemical medical device research. TMDs with high photothermal energy show promising results in applications in biomedicine, nanoelectronics, optoelectronics, photonics, and more.

TMDs have an ultra-thin structure (approximately 0.6–0.7 nm) with a high surface-to-volume ratio, which is an ideal structure for surface adsorption of loaded biomolecules as it provides an excellent platform for anchoring the adsorbents to the sensing site [9]. This large surface-to-volume ratio of TMDs increases sensitivity and selectivity while reducing biosensor power consumption. The increased density of surface sites allows for a higher probability of binding of the analyte to the site of the transducer elements of the sensors, which allows for manipulation of the optoelectronics and thus increases the sensitivity of the biosensors [10]. TMD-based sensors bypass the step of reactivity with the targeted biomolecule. The thickness of the TMD material determines the specific properties it contains. This layered structure also allows for the transport of charge carriers and the change in volume adjustment when ions are introduced, giving it a unique chemical composition and crystal structure. The flexibility of TMDs is an added advantage of TMDs, as it allows the manipulation of the material for the synthesis of devices at the point of use. This particular focus on the atomic thickness of transition metal dichalcogenides is sparking interest in research for next-generation electronic devices for medical applications [10].

Biosensors of interest can be divided into electrical, optical, and electrochemical biosensors. Electrical biosensors have a relatively simple and easy-to-use detection method that allows real-time detection of analytes. The binding of analytes to the TMD surface results in a current change signal that converts information about the analyte [3]. Optical biosensors are sensitive because they use surface plasmon resonance (SPR), which measures the local refractive index in response to the adsorption of the analyte of interest. Optical biosensors can also use fluorescence resonance energy transfer (FRET), which involves the transfer of non-radiant energy between two coupled fluorophores [11]. This review focuses on the electrochemical-based biosensing of target analytes by measuring interfacial impedance values once the analyte of interest is applied to the surface [12]. The electrodes in the biosensors include the working electrode (WE), the reference electrode (RE), and the counter electrode (CE). The electrical potentials are measured concerning the RE, while the CE makes the connections in the electrochemical cell. The WE can be suitably modified by nanomaterials to enhance the electrochemical activity, and biological detection elements can be used to detect analytes on the surface of the electrode. Analytes adsorbed on the surface of WE result in a unique electrochemical current or interfacial impedance response after modification [13,14]. The basic operating principle involves the determination of Faraday current by voltammetry or amperometry and the manipulation of interfacial impedance by electrochemical impedance spectroscopy [1].

**Figure 1:**
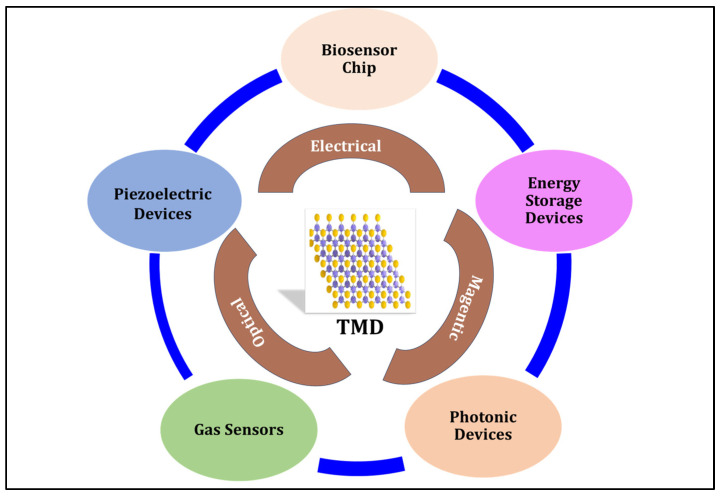
Overview of the structural and physical properties of transition metal dichalcogenides. Part of the figure (TMD structure) is from ref. [15].

Two-dimensional materials have promising potential for next-generation applications in energy storage, electronics development, sensor fabrication, and biomedical applications, as shown in Figure 1. Bioanalyte detection is critical for disease diagnosis, prognosis, and treatment and can be combined with the growing interest in 2D materials in the biomedical field [10,14]. The high surface-to-volume ratio and density of surface sites make 2D materials such as transition metal dichalcogenides favorable transducer elements in various biomolecular detection methods. Finite bandgaps and layer-dependent optoelectrical properties make TMDs superior to other known metals [9].

## 2. Structure and Properties 

In the last decade, we have witnessed the tremendous success of 2D materials, including graphene, and subsequently, transition metal dichalcogenides have received much attention due to their unique properties. Compared to graphene, TMDs exhibit a wide range of interesting properties, such as metallic (VSe_2_, NbS_2_), semimetallic (TiSe_2_, WTe_2_), semiconducting (WS_2_, MoS_2_), and insulating (HfS_2_) properties [16,17]. In addition, some TMDs exhibit distinguishable properties such as charge density waves, Mott transitions, and superconductivity. Interestingly, one can change the electronic properties of TMDs based on their layers (single or multilayer). Similarly, it is observed that the functional properties of TMDs can vary with the crystal structure depending on their atomic arrangement. The most common polymorphs are trigonal IT (single layers in the order AmC), hexagonal 2H (double layers in the order AmA CmC), and rhombohedral 3R (triple layers in the order AmA, BmB, CmC), where A, B, and C = chalcogenides and m = transition metal. TMDs in the 2H phase are known to be semiconducting and stable and have a large band gap in the visible region. TMDs in the 1T phase show higher reactivity compared to the 2T phase and are metallic or semimetallic in nature [17,18]. Randell nomenclature is generally used to name TMDs with a layered Van der Waals (vdW) crystal structure. In addition, the number of d-electrons can also influence the overall electronic properties of TMDs. Thus, the preferred coordination geometry and the Fermi energy (EF) depend on the orbital occupation. The elements of groups 5 and 6 prefer a trigonal–prismatic geometry, while the elements of groups 4 and 7–10 prefer an octahedral coordination. The only representative of group 8 is FeTe_2_, which was only recently discovered and whose research is still in progress, as shown by the small number of publications. Furthermore, the metallic or semiconducting nature of TMDs can be determined by the positioning of the Fermi energy. For example, the former has partially filled orbitals, and the EF lies within the band, while the EF lies in the energy gap in TMDs with partially filled orbitals [19,20].

From the above discussion, we conclude that the characteristic properties of TMDs are mainly determined by their elemental composition, bonding, coordination, and electronic configuration. Therefore, the controlled and facile synthesis of TMDs with desired properties remains a challenge.

## 3. Synthesis

**Figure 2:**
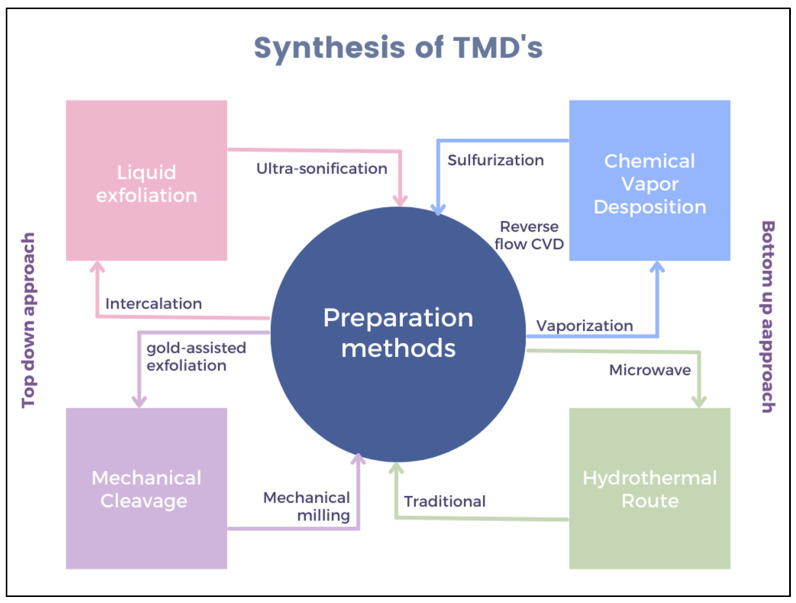
Different synthesis methods of TMD materials use top–down and bottom–up approaches. Part of figures from refs. [15,22,23,24].

TMD materials are used in various fields such as optoelectronic devices, gas sensors, environmental remediation, biosensors, energy storage, conversion, etc., due to their unique optical, electrical, and mechanical properties [1]. It is observed that intriguing and special properties can be uncovered by synthesizing thin-film TMDs in contrast to their bulk compounds. Moreover, both theoretical and experimental studies on semiconducting TMDs have revealed extraordinary properties, leading to new potential applications in the field of nanomaterials and nanodevices. Therefore, considerable efforts have been made in recent years to synthesize and develop high-quality TMDs. MoS_2_ and WS_2_ are the only TMDs that occur naturally in layered crystal form. Various methods have been used to obtain thin TMD layers, including mechanical/chemical exfoliation, chemical vapor deposition (CVD), intercalation, wet chemical methods, sonication, chemical vapor transport (CVT), and molecular beam epitaxy (MBE) [21]. These strategies can be roughly divided into top–down and bottom–up methods, as shown in Figure 2.

### 3.1. Top–Down Synthesis Methods

In the top–down synthesis route, TMDs are usually prepared by exfoliation, intercalation, and sonication. The low surface energy of many 2D TMDs (MoS_2_, WS_2_, and NbSe_2_) allows for easy exfoliation. The applied mechanical force splits/separates the individual layers of the source material into thin layers. The most widely used, efficient, and basic method is the scotch tape method, which overcomes the vdW attraction by applying adhesive forces [25]. However, it is extremely difficult to ensure structural integrity, control of flake size and thickness, and transfer of TMDs after synthesis if this method is to be optimized for industrial applications. Apart from the limited commercial availability of bulk TMDCs, defect-free exfoliation requires considerable effort and suffers from low reproducibility. Nevertheless, mechanical exfoliation is still considered the most popular method for synthesizing single-layer TMDs with high crystal quality and excellent electronic and optical properties. Bellus et al. used the mechanical exfoliation route to synthesize a MoSe_2_/WS_2_ heterostructure with a trion binding energy of ~62 meV [26]. Another technique is to introduce alkali metal ions for intercalation between layers and further exfoliation. However, alkali metals tend to aggregate, which is time-consuming, and this method detects impurities from the outside. Furthermore, chemical exfoliation can lead to the formation of additional intermediates with undesirable physical and chemical properties. A recent study on Li interaction as an attractive route for large-scale synthesis of single-layer MoS_2_ films was presented by Eda and co-workers. In the study, it was reported that exfoliation by Li intercalation resulted in a loss of the original semiconducting properties of MoS_2_ due to structural changes [27]. Similarly, controlled electrochemical intercalation of Li in bulk TMDs was used to synthesize a few layers of thick nanosheets of NbSe_2_, WSe_2_, Sb_2_Se_3,_ and Bi_2_Te_3_ [28]. Mao et al. developed ultrathin 2D nanosheets of WSe_2_, MoSev, WS_2_, and MoS_2_ with a thickness of ∼3 nm and a lateral size of a few hundred nanometers by an ultrasound-assisted synthesis route. They concluded that mild liquid-phase exfoliation of bulk TMDs is a gentle exfoliation method for obtaining high-quality TMD crystals compared to other conventional methods [7].

### 3.2. Bottom–Up Synthesis Methods

In the bottom–up method, molecules are used as starting reagents in suitable deposition techniques and can be further divided into multicomponent or single-source precursor systems. This method mainly includes wet chemical synthesis, CVD, and MBE. The wet chemical synthesis method allows the controlled growth of TMD nanosheets. Generally, metal salts are used as starting materials for nanosheet synthesis. Wet chemical synthesis can be roughly divided into hydrothermal and solvothermal methods. In the hydrothermal method, the reaction takes place in a sealed autoclave at elevated temperature and pressure under an inert gas atmosphere. The solvothermal method is very similar to the hydrothermal method, with the only difference being that an organic solvent is used instead of water. This method also requires a sealed vessel with a reaction temperature that is above the boiling point of the solvent used. When the temperature rises above the boiling point of the solvent, the formation of TMD crystals occurs under high pressure. As a representative example, Lee et al. have shown the synthesis of MoS_2_ TMDs with a 1T edge using dimethylformamide (DMF) as a solvent [29].

CVD is another bottom–up technique used in the synthesis of TMDs. In this process, 2D TMDs are grown on a substrate by a reaction between the thermal evaporation of a metal precursor and thermally evaporated chalcogen elements such as selenium or sulfur. The CVD process is a powerful method for developing single or double-layer 2D materials with large dimensions. The technique offers high-quality TMD layers with controllable layer numbers, domain sizes, and excellent properties, as well as a simple and practical solution for industrial requirements. Despite these advantages, the quality of the obtained single-layer TMDs has serious drawbacks that hinder their application in nanotechnology. Moreover, CVD requires stringent conditions, such as elevated temperatures during synthesis, which leads to numerous crystallographic defects and impurities. Researchers reported the synthesis of centimeter-sized continuous films from single-layer MoS_2_ films directly on Si substrates with an oxide layer, also achieving large domain sizes of more than 20 μm within the films. This is achieved by orienting the growth substrate vertically to improve the uniformity of the starting material compared to horizontally oriented growth substrates [11]. Similarly, molecular beam epitaxy (MBE) is one of the first scalable methods for the synthesis of TMDs. The MBE method requires an ultra-high vacuum (UHV) with elements of high purity. The elements are heated in an effusion cell of electron beam evaporators until they slowly begin to sublime. The thermally evaporated gaseous elements react with each other and condense on the substrate. This technique enables the growth of ultra-thin crystalline heterostructures with high yields. Moreover, the crystallinity of the obtained TMD is monitored in situ by reflecting high-energy electron diffraction and low-energy electron diffraction. Epitaxial layers of WS_2_ using MBE are discussed, for example, by Jaeger Mann and his co-workers [30].

In addition to these techniques, there are a variety of other methods, such as the polymer-assisted transfer method, pulsed layer deposition, cation exchange, etc., that are used to develop TMDs. Finally, the TMDs synthesized by the direct route may not work well in biosensor applications. Therefore, surface modification of TMDs is a suitable method to improve their performance. Various materials, such as carbon-based materials, polymers, proteins, metal nanoparticles, MOFs, MXenes, etc., can be associated with TMDs. The main objective of performing this surface modification is to achieve improved electrochemical properties, biocompatibility, and low toxicity.

## 4. Electrochemical Biosensor and Application of TMDs for Detection of Biomolecules

**Figure 3:**
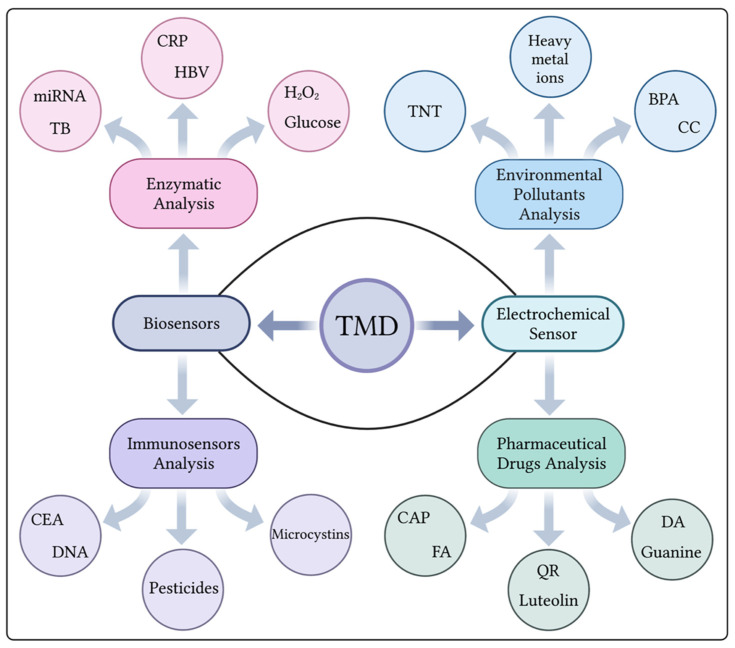
Applications of TMD materials for the detection of different analytes using various biosensing strategies. (Created using Biorender.com (accessed on 3 November 2023)).

TMDs have become increasingly popular due to their high volume-to-surface-area ratio, making them an attractive choice in various fields, including clinical applications. One particular area of interest is their use in biosensors, particularly electrochemical biosensors. In this type of biosensor, TMDs are utilized to capture target molecules that are essential to vital processes and substances that can affect life. As mentioned in Figure 3, immunosensors are used to detect target molecules such as carcinoembryonic antigens (CEA), DNA, pesticides, and forms of microcystins, while enzymatic sensors are used to detect molecules in the body such as miRNA, CRP, glucose, and H_2_O_2_. Electrochemical sensors offer additional possibilities for measuring biological molecules such as luteolin and guanine, as well as biological molecules in various pathological conditions such as Friedreich’s ataxia (FA). The other aspect related to electrochemical detection has also been reported for the detection of environmental pollutants using TMDs, especially TNT, BPA, and heavy metal ions [31]. Although TMDs have such great potential, this review article focuses specifically on the methods of electrochemical detection of biological components. Transition metal dichalcogenides (TMDs), a class of graphene-like 2D materials, have attracted considerable attention in the field of electrochemical sensing. Among the various TMDs, MoS_2_ stands out as the most intensively studied and widely recognized material for its sensing capabilities [32]. Although other TMDs exist, MoS_2_ has become the main focus of research due to its remarkable performance in electrochemical sensing applications. In this review article, we mainly focus on the sensing strategies that have been reported for biomolecule detection using TMD-based materials as interfaces in electrochemical biosensing. For the better understanding of the reader, we have tried to divide the biomolecules into simple and complex molecules according to their structural complexity.

**Figure 4:**
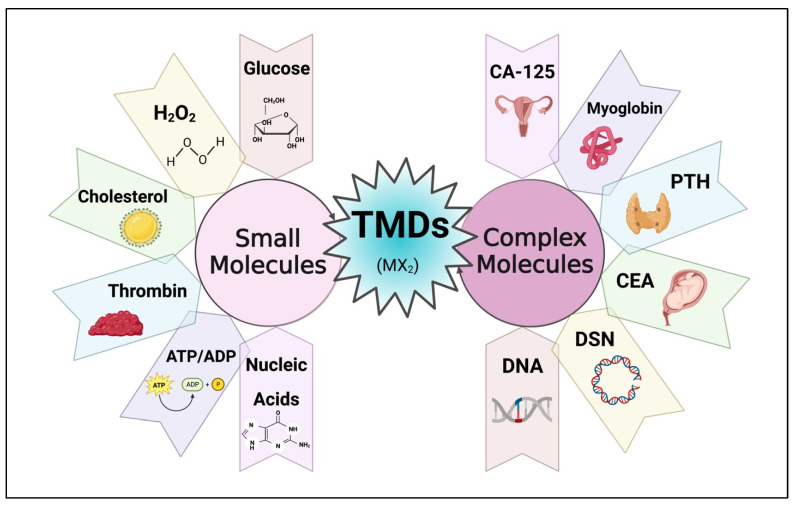
Utilization of TMD materials for electrochemical biosensing of small and complex molecules. (Created using Biorender.com (accessed on 3 November 2023)).

TMDs have proven to be excellent candidates for electrochemical sensors, offering advantages in both simplicity and versatility. These TMDs provide a reliable platform for the detection of various molecules, including both simple and complex analytes, as depicted in Figure 4. Their large surface area enables not only efficient but also accurate detection of target molecules, resulting in higher sensitivity and selectivity. Furthermore, the unique electronic properties of MoS_2_ contribute to its superior electrocatalytic activity, which enables highly sensitive detection [33]. In addition to TMD’s ability to detect simple molecules, it demonstrated its potential as a biocompatible matrix for the immobilization of biomolecules. These specific properties of TMD materials enabled the development of biosensors that can detect complex molecules but also have properties that allow them to capture even small biomolecules. By integrating specific enzymes or antibodies, these biosensors can selectively recognize biomolecules and detect the target biomolecules with sensitivity and precision. The combination of TMD’s biocompatibility with the catalytic properties of enzymes creates a powerful platform for the detection of complex analytes [34]. Although there are many TMDs within the family of graphene-like 2D materials, MoS_2_ remains the most researched and extensively studied material due to its superior sensing capabilities. The unique properties of MoS_2_, combined with its frequent availability and ease of synthesis, have helped it become a popular research focus in electrochemical sensing.

### 4.1. Small Molecules

Research has made great strides in the development of sensors, with MoS_2_ nanosurfaces, in particular, being used for the precise detection of small molecules. These sensors promise promising applications in a wide range of fields, including healthcare, environmental monitoring, analytical sciences, and beyond. Taking advantage of the exceptional properties of TMDs, scientists have developed sensors that feature high performance with excellent selectivity and enhanced sensitivity. TMDs also proved successful in the detection of biomolecules with excellent reliability and can be specifically tailored for small-molecule detection. Moreover, MoS_2_ nanosurfaces, in combination with carbon nanotubes (CNTs) and graphene (GR), have played a crucial role in the electrochemical detection of various small molecules. The integration of MoS_2_, CNTs, and GR provides a conductive and stable platform for precise detection that enables accurate monitoring of small molecule concentrations in biological samples.

The remarkable progress made with MoS_2_ nanosurfaces in detecting small molecules underlines their immense potential for diagnostic and analytical purposes. By taking advantage of the unique properties and benefits of MoS_2_, scientists have developed sensors with promising applications in disease diagnosis and biomedical research. Further research and development in this area holds the potential for even greater sensitivity and specificity, paving the way for a wide range of future small-molecule detection applications [35]. Detection methods can also be adapted for the detection of specific molecules.

Reduced graphene sensors and Ni-doped sensors have proven to have excellent electrocatalytic performance, which helps in improving the linear range of sensors. Researchers have been able to effectively perform oxidation studies of glucose and have achieved lower detection limits and longer stability. Building on the unique properties of Ni-doped MoS_2_ and reduced graphene oxide (rGO), researchers have developed sensors that provide a highly reliable and sensitive platform for monitoring glucose levels. These sensors feature excellent electrocatalytic capabilities, a broad linear range, minimal detection limits, and outstanding stability. By utilizing the catalytic power of Ni-doped MoS_2_ and the conductivity of rGO, precise and accurate glucose detection becomes possible. Other commonly used TMD-based detection methods for small molecules, such as glucose, include amperometry [36], photoelectrochemical (PEC) detection [37], and sequential cyclic voltammograms (CVs) [38]. Amperometry offers the following advantages: the dispersion of Cu_2_O nanoparticles on MoS_2_ nanosheets, the porous 3D structures, and the surface phase transitions between Cu_2_O and MoS_2_ could enable efficient electron transfer at the interface [36]. PEC is unique in that when sunlight generates electrons and holes, they are directed to the cathode and anode, respectively, due to the separation between the excitation source (light) and the detection signal (photocurrent) [37,39]. 

Researchers have explored different materials for the detection of glucose and evaluated their effectiveness using different techniques. Two-dimensional transition metal dichalcogenides have also shown great promise for electrochemical biosensors. Tungsten dichalcogenides such as WS_2_ and WSe_2_ have shown highly efficient heterogeneous electron transfer capabilities compared to molybdenum dichalcogenides (MoS_2_ and MoSe_2_), leading to higher electrochemical responses in glucose biosensors [40]. In addition, the glucose-sensing properties of exfoliated TMDs, particularly MoS_2_, MoSe_2_, and MoTe_2_, were investigated, and all three materials exhibited improved electrical conductivity and met analytical requirements for glucose detection. MoS_2_-based biosensors showed higher specificity and lower detection limits than MoSe_2_ and MoTe_2_ [41]. These results highlight the potential of different materials, such as non-transition metal chalcogenides, TMDs, and their nanocomposites, for glucose detection and biosensor applications.

Another interesting area is the detection of hydrogen peroxide (H_2_O_2_), an important small molecule involved in numerous biological processes. MoS_2_-based sensors have proven useful for the accurate and selective detection of H_2_O_2_. Various sensor designs have been explored, including bimetallic electrocatalysts, enzymatic nanocomposites, and metalloprotein-based sensors. These sensors exhibit remarkable sensitivity and selectivity and allow precise measurements of H_2_O_2_ concentration in complex biological samples. Integrating MoS_2_ with complementary materials enhances sensor performance by creating additional binding sites and further improving electron transfer kinetics. Many of these techniques overlap with those for other small molecules, but for each type of small molecule, there are commonly used methods. For glucose and hydrogen peroxide, amperometry is generally used [42], but for H_2_O_2_, chronoamperometry is most commonly used [43]. In chronoamperometry, a rectangular potential is applied to an electrode, and the current of the electrode is measured as a function of time [44].

**Figure 5:**
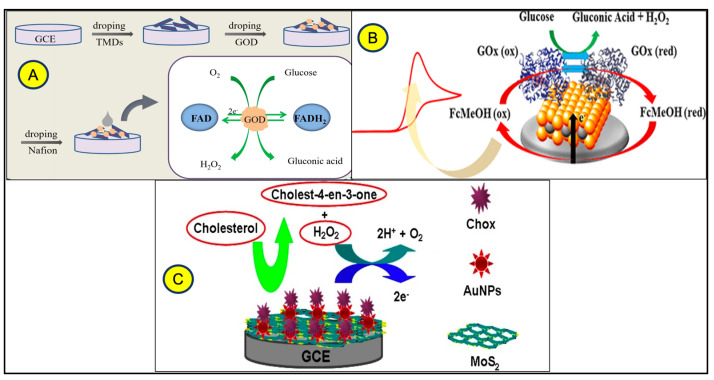
Utilization of TMDs for small molecules using electrochemical detection methods of glucose (**A**) re-printed from ref. [45], (**B**) ref. [40], and cholesterol (**C**) ref. [46] using TMDs.

MoS_2_ is widely reported for the detection of glucose in the absence [45] and presence [40] of redox molecules (Figure 5A,B). The detection of cholesterol, an important small molecule associated with cardiovascular disease, has also attracted considerable attention in TMD sensor research. Scientists have developed sensors containing MoS_2_, gold nanoparticles (AuNPs), and cholesterol oxidase (Chox) to address the need for accurate cholesterol monitoring, which is important in many diseases and conditions. These sensors offer exceptional catalytic response, reliable recoveries in spiked samples, and are suitable for point-of-care diagnosis of cholesterol-related diseases. The compatibility between MoS_2_ and AuNPs enhances the performance of the sensor by increasing the number of catalytic sites and improving the efficiency of electron transfer. The method used for cholesterol detection is amperometry, which has already been explored for a variety of small biomolecules [46] (Figure 5C).

With the advent of the use of TMD for the detection of molecules in the human body, other techniques have been explored that are not specific to one molecule and have robust binding capacity for a variety of biomolecules. Another method being discussed for the detection of small molecules and proteins is the use of MoS_2_ films. This method essentially uses electrochemically reduced MoS_2_ nanosheets with other electrochemically active materials to measure biomolecules such as glucose and dopamine. Not only is the detection of a single analyte possible, but multiplex detection has also been performed, e.g., of uric acid, ascorbic acid, and dopamine, using a system based on gold nanoparticles and MoS_2_ nanocomposites [47]. Other molecules that have been explored using TMD nanosheets with electrochemical detection are thrombin, adenosine triphosphate, guanine, and adenine. Another method for detecting dopamine is differential pulse voltammetry (DPV) [48]. In DPV, the sample containing dopamine is subjected to a series of voltage pulses. The current response produced by these pulses is then measured and analyzed. DPV is particularly effective because it filters out unwanted background currents, allowing for particularly high sensitivity. Mn-MoS_2_ deposited on a plate of pyrolytic graphite has been shown to increase detectability by improving charge transfer. By analyzing the current response, the dopamine concentration can be determined. The Mn-MoS_2_-based sensor provides excellent selectivity for dopamine, even in the presence of interfering substances [47]. This technique opens possibilities for non-invasive monitoring of dopamine levels in body fluids such as sweat and is, therefore, promising for future wearable diagnostic devices [49].

### 4.2. Complex Molecules

The field of analytical chemistry has made remarkable progress in recent years with the development of electrochemical biosensors for the detection of complex biomolecules. These biosensors have revolutionized the way we analyze and understand different biomolecules by providing a highly sensitive and selective approach. A particular focus is the detection of specific nucleic acid sequences, which provide valuable insights into diseases at the molecular level, especially nucleic acids and proteins, lipids, and carbohydrates.

Researchers have made significant progress by using MoS_2_ nanostructures as efficient platforms for detecting complex nucleic acid structures. These nanostructures have unique properties, such as a large surface area and excellent biocompatibility, making them perfect for immobilizing DNA probes. By combining these probes with advanced techniques such as duplex-specific nucleases (DSNs), enzymatic signal amplification, and electrochemical redox cycle systems, scientists have been able to achieve an impressive level of sensitivity and selectivity. This also allows them to detect nucleic acid abnormalities, genetic mutations, and changes in gene expression, which are crucial for the diagnosis and monitoring of various diseases, including cancer [50].

Two-dimensional TMDs have also been used in the detection of more complex molecules, such as DNA or long nucleic acid chains, for the detection of specific chains. One method for this detection was explored using MoS_2_ nanoflakes. This method for large-scale detection of DNA sequences consists of three components: the irreversible oxidation of these nanoflakes, the immobilization of the sample single-stranded DNA (ssDNA) probes on a carbon electrode, and the hybridization of the complementary ssDNA strand with the immobilized probes. This results in measurable voltammetric signals between the affinity of the MoS_2_ nanoflakes and the sample strand, which are then used to determine the presence of the target DNA strand [48]. DPV is one of the most common methods for DNA detection [51,52]. DPV has also been mentioned in dopamine detection, making it a technique that can be further explored for both simple and complex molecules, even miRNAs and ctDNA [50,53,54,55,56].

**Figure 6:**
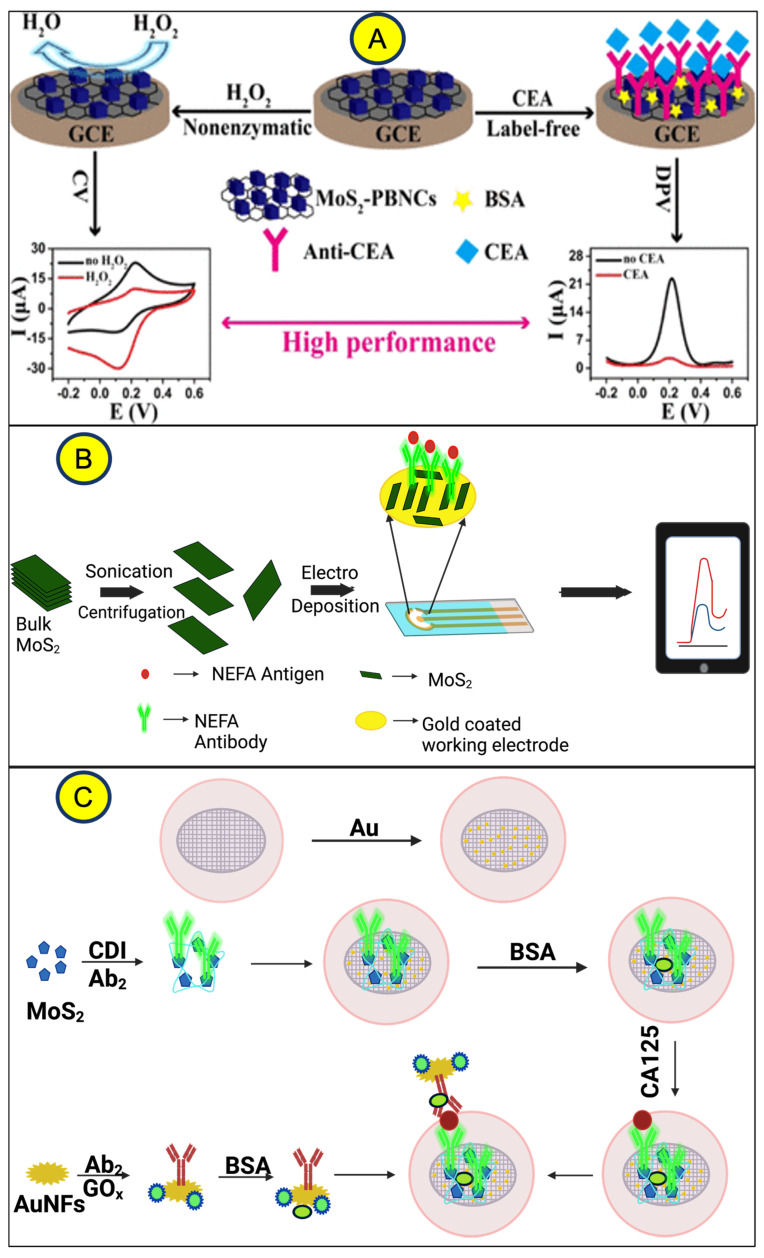
Detection of complex molecules CEA, NEFA, and CA215 using MoS_2_. Reprinted from (**A**) ref. [57], redrawn from (**B**) ref. [59], and (**C**) ref. [12] using Biorender.com (accessed on 3 November 2023).

In addition, electrochemical biosensors have also demonstrated their ability to detect protein biomarkers. As shown in Figure 6, MoS_2_ nanostructures can detect a wide range of protein markers, from cardiac markers such as myoglobin (Mb) to tumor markers such as cancer antigen-125 (CA-125) and carcinoembryonic antigen (CEA) [53,57]. By modifying the biosensor surfaces with specific antibodies, researchers can selectively detect these biomarkers. The level of electrochemical response generated by the biosensors is directly related to the concentration of the targeted biomolecules and allows for precise quantification of these molecules. This interesting feature of electrochemical TMD detection holds enormous potential for clinical diagnosis, as it enables early detection of disease and facilitates personalized treatment strategies. Another protein being explored is riboflavin-binding protein, CA-125 [12], and CEAs are also commonly detected by various forms of voltammetry (DPV), which is beneficial because it can distinguish analytes with similar oxidation potentials [58].

Also promising is the exploration of combining MoS_2_ nanostructures with other materials, such as ZnO nanosheets, which has expanded the possibilities of electrochemical biosensing. This hybrid approach allows researchers to detect complementary DNA sequences associated with specific genetic mutations, providing crucial information and advantages for the diagnosis of genetic disorders and diseases such as acute promyelocytic leukemia. A simple ultrasound exfoliation method was used to synthesize MoS_2_ sheets and construct a DNA-based biosensor to detect guanine and adenine [58]. By incorporating MoS_2_ nanoflowers into biosensors, scientists have achieved highly sensitive detection of complex protein markers such as thrombin [60] and parathyroid hormone (PTH) [41] by voltammetric methods such as cyclic voltammetry (CV) and DPV. These biosensors offer both excellent reproducibility and regeneration capabilities, making them practical for use in real clinical settings.

The detection of fatty acids using 2D-MoS_2_ and possibly other TMD nanosheets and voltammetry, an electrochemical technique, is also being investigated. NEFA antibodies were bound to the nanosheets to allow specific binding, such as in DPV sensing methods [57]. The resulting immunosensor exhibited a wide detection range and reliable performance, promising applications for on-farm fatty acid monitoring and livestock improvement. These techniques have proven useful not only in livestock but also in human medicine and point-of-care applications.

The development of electrochemical biosensors for the detection of complex biomolecules represents a breakthrough in analytical chemistry. These biosensors serve as powerful tools for deciphering the molecular aspects of disease and hold immense potential for early diagnosis, personalized medicine, and treatment monitoring. The integration of TMD nanostructures, with their unique properties and compatibility with biological systems, has played a critical role in improving these biosensors’ sensitivity, selectivity, and overall performance. Ongoing research and innovations in this field promise more exciting advances that will lead to better healthcare outcomes and a deeper understanding of the intricate workings of biological processes [35]. Table 1 shows the recent research reports using MoS_2_ for the detection of different biomolecules.

## 5. Future Perspectives

The rapid development of nanotechnology has enabled the wide use of various 2D technologies in various biomedical fields, such as biosensing, medical imaging, drug delivery, etc. Due to the excellent electrochemical properties of TMDs, this review aims at their role in electrochemical biosensing. Despite enormous research conducted on the TMDS, their application in the biomedical domain still remains largely unexplored. Currently, a major gap required to address TMDs is related to the wafer-scale synthesis and fabrication processes integrated with state-of-the-art techniques. One of the critical challenges for TMDs to compete with existing technologies is devising a low-cost wafer-scale synthesis solution. A uniform wafer-scale synthesis is an essential criterion for mass production; otherwise, the device-to-device variation could forestall the way for a PoC sensor for consumers. Compared to 0D and 1D materials, the planner structure of 2D TMDs makes them inherently compatible with the existing state-of-the-art fabrication technologies for biosensors. Synthesis methods, such as CVD, MOCVD, and ALD, have the potential for high-quality synthesis and minimal device-to-device variation. The combination of biosensors with noble metal and semiconductor nanomaterials can achieve sensitive and accurate detection of target analytes, improving the sensitivity and selectivity of detection and reducing the response time of signals by doping and modifying different substances with noble metal and semiconductor nanomaterials. Based on the phenomena of plasma resonance and surface Raman spectroscopy enhancement, researchers can also continue to develop many optical biosensors with higher accuracy. The gradual maturation of noble metal and semiconductor nanomaterials provides new insight for research on electrochemical sensors. In the future, with the progress of synthesis technology for nanomaterials and the discovery of new metal-based nanomaterials, the following are the research directions worth exploring for biosensors: (1) Set out to develop sensors that are simple, convenient, and can respond quickly in natural conditions. (2) Taking advantage of the properties of noble metal and semiconductor nanomaterials (surface effect, small size, macroscopic tunneling effect) to build micron-sized sensors suitable for intracellular use. In addition, attention can also be paid to constructing a bionic interface, which is conducive to the study of neural activity. (3) Biosensors can be used to detect life-threatening diseases in the heart, brain, kidneys, and other critical parts of the body. Therefore, the development of flexible, wearable electrochemical biosensors has broad prospects. Due to the presence of interference in human body fluids (such as glucose, lactic acid, and metal ions), the specific detection of target substances is particularly critical. By combining with other high-quality materials, noble metals and semiconductor nanomaterials can realize the specific recognition of the detection substance and reduce the detection limit. State-of-the-art PoC products based on TMDs have not been commercialized. There is a need for robust study regarding wafer-scale synthesis, fabrication processes, and integration with state-of-the-art modern electronic fabrication. One of the critical challenges for TMDs to compete with existing technologies is devising a low-cost wafer-scale synthesis solution. A uniform wafer-scale synthesis is an essential criterion for mass production; otherwise, the device-to-device variation could forestall the way for a PoC sensor for consumers. Compared to 0D and 1D materials, the planner structure of 2D TMDs makes them inherently compatible with the existing state-of-the-art fabrication technologies for biosensors. Synthesis methods, such as CVD, MOCVD, and ALD, have the potential for high-quality synthesis and minimal device-to-device variation. TMDs are suitable for new-generation wearable healthcare devices with their high mechanical flexibility and stability. They are generally grown at high temperatures, but flexible polymer substrates are incompatible with direct growth. TMDs are transferred from the growth substrate to the device substrate. Currently, ultrathin TMDs are transferred using polymethyl methacrylate (PMMA) based on wet chemical methods [25]. The transfer samples contain PMMA residue, which creates defects and wrinkles. There is a big challenge for efficient wafer-scale transfer of 2D TMDs without creating defects or wrinkles. Thus, an improved transfer process is needed for highly efficient device fabrications or to find a solution for low-temperature growth directly on the flexible substrate. As part of the sensing application, functionalization of the TMD surface is essential for highly selective biosensors and minimizing false-positive responses. There is a need for efficient surface engineering for the attachment of bioreceptors. Enormous work has been conducted during the last decade, and further improvements for suitable surface modification are still needed. Once the scientific community resolves the present technological challenges, the 2D-TMD-based sensing platform has an excellent opportunity for next-generation personalized healthcare devices. With their mechanical flexibility, ultrathin thickness, and optical transparency, 2D-TMDs have the potential to be incorporated into textiles for continuous monitoring of health conditions. Two-D TMDs have already demonstrated label-free detection of bioanalytes down to aM concentration, much lower than many current technologies, suggesting tremendous potential for next-generation personalized sensing platforms. The scientific community has made enormous progress in developing 2D TMD-based materials for biosensing applications in the last ten years. Further development and realizations are needed for sustainable academic research and collaboration with industrial partners to achieve next-generation applications in personalized health monitoring, wearable technologies, and low-power, portable diagnostics with superior performance compared to existing technologies.

## 6. Conclusions

Since transition metal dichalcogenides have emerged from the shadow of graphene in terms of improved properties in electrochemical biosensing applications, many biomedical applications have been explored. These two-dimensional TMDs are still relatively unexplored in the field of biosystems, as their fundamental interactions with cell organelles are less explored compared to conventional materials. The above-mentioned properties of transition metal dichalcogenides have led to the exploration and development of next-generation biosensor applications. The detection of biomarkers, which play an important role in clinical diagnostic methods, is essential for disease diagnosis, the status of disease progression, and treatment plans. Biosensors should ideally be able to detect low levels to detect disease early, leading to a promising treatment and recovery plan. Two-dimensional TMDs have a high density of active sites on the surface, resulting in a highly sensitive but very low detection limit. Surface modification is a promising area of research for TMDs, as methods to functionalize the surface, corners, and edges of biosensors can be useful for biomedical applications. Ultimately, the emerging field of two-dimensional transition metal dichalcogenides has a high potential for commercial and industrial bio-applications and deserves significant attention and investment.

## Figures and Tables

**Table 1:** Recent reports on electrochemical detection of small and complex molecules using TMDs.

Material	Detection Method	Target Biomolecule	Buffer/Biofluid	Detection Range	Reference
WS_2_/Graphite	CV	Adenine/Guanine	PBS	0.5–20 µM	[61]
MoS_2_, TaS_2_, and TiS_2_	FL	DNA	PBS	0–20 nM	[62]
N-TiO_2_	CV	DA	serum	0.003–300 μM and 1 nM	[63]
CeO_2_	CV	CRP	serum	0.3 to 7.0 mg L^−1^	[64]
WS_2_–Gr	CV	DNA	serum	0.01 to 500 pM	[65]
WSe_2_	voltammetric	stx1 and stx2	urine, serum, milk	50 pg mL^−1^ to 100 ng mL^−1^	[66]
MoSe_2_/MoS_2_	DPV and CV	miRNA-155	blood	1 fM to 1 nM	[67]
AuNPs-TiS_2_	CV	uricase	serum	5–2000 µM	[68]
VS_2_	DPV	ssDNA	PBS	of 5.0 × 10^−13^–5.0 × 10^−10^ M	[69]
WeS_2_	PEC	alpha-synuclein	serum	10 aM to 1 nM	[70]
MoS_2_-graphene (MG)	CV	PTH	serum	1.0 and 50.0 pg/mL	[3]
VS_2_	DPV	17β-estradiol	urine	6 × 10^−14^ M and 5 × 10^−13^ to 5 × 10^−9^ M	[71]
WS_2_	CV, DVP, EIS	prothrombin	serum	100 fg mL^−1^ to 100 ng mL^−1^	[72]

## Data Availability

No data sources are available.
